# Current Trends and Challenges in the Clinical Translation of Nanoparticulate Nanomedicines: Pathways for Translational Development and Commercialization

**DOI:** 10.3389/fphar.2018.00790

**Published:** 2018-07-17

**Authors:** Susan Hua, Maria B. C. de Matos, Josbert M. Metselaar, Gert Storm

**Affiliations:** ^1^Therapeutic Targeting Research Group, School of Biomedical Sciences and Pharmacy, University of Newcastle, Callaghan, NSW, Australia; ^2^Hunter Medical Research Institute, New Lambton Heights, Newcastle, NSW, Australia; ^3^Department of Pharmaceutics, Utrecht Institute for Pharmaceutical Sciences, Utrecht University, Utrecht, Netherlands; ^4^Department of Biomaterials Science and Technology, MIRA Institute for Biomedical Technology and Technical Medicine, University of Twente, Enschede, Netherlands; ^5^Department of Experimental Molecular Imaging, RWTH University Clinic Aachen, Aachen, Germany; ^6^Imaging Division, University Medical Centre Utrecht, Utrecht, Netherlands

**Keywords:** nanomedicine, nanoparticles, drug delivery systems, clinical translation, challenges, commercialization, biological, regulations

## Abstract

The use of nanotechnology in medicine has the potential to have a major impact on human health for the prevention, diagnosis, and treatment of diseases. One particular aspect of the nanomedicine field which has received a great deal of attention is the design and development of nanoparticulate nanomedicines (NNMs) for drug delivery (i.e., drug-containing nanoparticles). NNMs are intended to deliver drugs via various mechanisms: solubilization, passive targeting, active targeting, and triggered release. The NNM approach aims to increase therapeutic efficacy, decrease the therapeutically effective dose, and/or reduce the risk of systemic side effects. In order to move a NNM from the bench to the bedside, several experimental challenges need to be addressed. This review will discuss the current trends and challenges in the clinical translation of NNMs as well as the potential pathways for translational development and commercialization. Key issues related to the clinical development of NNMs will be covered, including biological challenges, large-scale manufacturing, biocompatibility and safety, intellectual property (IP), government regulations, and overall cost-effectiveness in comparison to current therapies. These factors can impose significant hurdles limiting the appearance of NNMs on the market, irrelevant of whether they are therapeutically beneficial or not.

## Introduction

**Figure 1:**
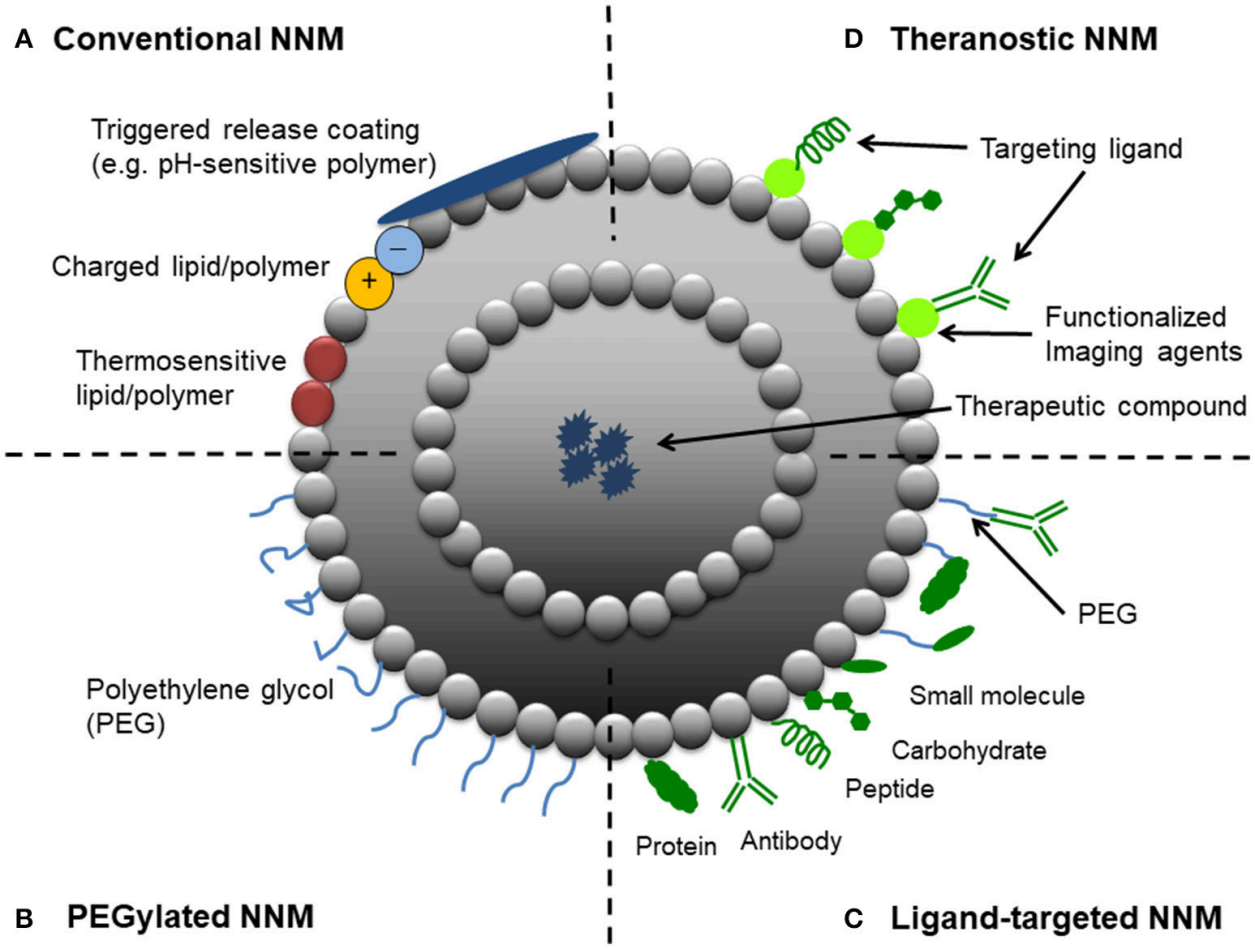
Schematic representation of different strategic designs for nanoparticulate nanomedicines (NNMs). **(A)** Conventional NNM—These NNMs can be modified with charged lipids/polymers, thermosensitive lipids/polymers and/or components for triggered release (e.g., pH-sensitive coating). **(B)** PEGylated NNM—Nanoparticle characteristics and behavior *in vivo* can be modified by the addition of a hydrophilic polymer coating, polyethylene glycol (PEG), to the NNM surface to confer steric stabilization. **(C)** Ligand-targeted NNM—Nanoparticles can be used for active targeting by attaching ligands (e.g., antibodies, peptides and carbohydrates) to its surface or to the terminal end of the attached PEG chains. **(D)** Theranostic NNM – These NNM systems consist of an imaging component and a therapeutic component, and may include a targeting element.

Nanomedicine applies nanotechnology to highly specific medical interventions for the prevention, diagnosis, and treatment of diseases (Teli et al., 2010). In the last several decades, the application of nanotechnology for medical purposes has received significant attention from researchers, academia, funding agencies, government, and regulatory bodies (Allen and Cullis, 2004; Sercombe et al., 2015; Hare et al., 2017). One particular aspect of the nanomedicine field which has received a great deal of attention is the design and development of nanoparticulate nanomedicines (NNMs) for drug delivery (i.e., drug-containing nanoparticles), which are most often given by parenteral (particularly intravenous) administration. NNMs are intended to increase the therapeutic index of drugs (i.e., increase efficacy and/or reduce toxicity) by delivering them via various mechanisms: solubilization, passive targeting, active targeting, and triggered release (Figure 1). Nanoencapsulation gives the opportunity to protect fragile compounds that degrade easily in biological environments and to provide solubilization, i.e., to deliver compounds which have physicochemical properties that strongly limit their aqueous solubility and therefore systemic bioavailability (Talekar et al., 2015; Kim et al., 2016; Larsson et al., 2017; Mishra et al., 2017; Shajari et al., 2017). Targeted drug delivery and triggered release of NNMs have been shown to be beneficial for increasing the therapeutic index of compounds, by improving the *in vivo* fate of drug molecules such that more efficient delivery to the target site is achieved (to yield improved therapeutic effects) with less accumulation in many healthy body sites (to reduce toxicity). Also NNMs have been studied for their ability to stimulate target cell uptake and improve intracellular trafficking, processes sometimes required when they have localized in target tissues (Mastrobattista et al., 1999; Hua, 2013; Hua et al., 2015).

Although NNMs have demonstrated significant therapeutic advantages for a multitude of biomedical applications, their clinical translation has not progressed as rapidly as the plethora of positive preclinical results would have suggested (Luxenhofer et al., 2014). In order to move a NNM from the bench to the bedside, several experimental challenges need to be addressed. From a biological perspective, these include studies focused on understanding the *in vivo* fate and interactions of NNMs with the blood, tissue, cellular, and intracellular compartments in the host in healthy and diseased states (Nehoff et al., 2014; Sercombe et al., 2015; Hare et al., 2017). For NNMs to have clinical translation potential, the complexity in their design and development also needs to be minimized as much as possible to create systems that are able to be reproducibly prepared and characterized (Lammers, 2013; Barz et al., 2015). This review will address the current trends and challenges in the clinical translation of NNMs as well as the potential pathways for translational development and commercialization.

## Current trends in the clinical translation of nanomedicines

NNMs are often studied to improve drug targeting to specific sites of disease (i.e., site-specific drug delivery) and/or attenuate localization in healthy non-target tissues (i.e., site-avoidance drug delivery; Rizzo et al., 2013). The vast majority of NNMs in preclinical and clinical development as well in clinical use are for targeting a wide variety of cancers and tumors (Hare et al., 2017). The application of NNM-based therapies for drug targeting to non-cancer conditions has increased in recent years. In particular, NNMs have been developed to address the clinical challenge of effectively managing inflammatory diseases by exploiting the underlying biology of these conditions (Milane and Amiji, 2017). Non-cancerous inflammatory diseases that have been explored with NNM therapy include rheumatoid arthritis, inflammatory bowel disease, asthma, multiple sclerosis, diabetes, and neurodegenerative diseases (Milane and Amiji, 2017).

### Enhanced permeability and retention (EPR) effect and passive accumulation of NNMs

The EPR effect refers to the preferential localization of NNMs in pathological tissues due to the enhanced permeability of the vasculature that supplies such tissues (e.g., tumors and inflammatory conditions). Deregulations in angiogenesis and/or the increased expression and activation of vascular permeability factors predominates at these sites, which can lead to fenestrations allowing passage of NNMs (Hashizume et al., 2000; Nehoff et al., 2014). In addition to the enhanced leakiness of tumorous and inflamed blood vessels, the EPR effect also relates to the observation that solid tumors tend to lack functional lymphatic drainage, which limits the removal of extravasated NNMs from the target site (Maeda et al., 2013; Danhier, 2016). These pathological properties allow NNMs to accumulate at pathological sites and is referred to as passive targeting. To achieve this, it is important that NNMs with drug cargo circulate long enough in the bloodstream (i.e., show prolonged circulation kinetics). This can be achieved by conjugating polyethylene glycol (PEG) to the surface of NNMs (Figure 1). Thus, the EPR effect is expected to increase the therapeutic efficacy of NNMs in comparison to small molecules, which often show inferior pharmacokinetic properties (Matsumura and Maeda, 1986; Hobbs et al., 1998; van der Meel et al., 2013). The EPR effect was first observed in 1986 (Matsumura and Maeda, 1986) and has since been exploited particularly for the development of NNMs for passive tumor targeting, leading to NNMs with adequate physicochemical properties and prolonged circulation half-life that accumulate in tumors over time (Maeda et al., 2013; Nakamura et al., 2015; Danhier, 2016). The EPR effect and thus extent of passive targeting is highly dependent on the tumor pathophysiology. Currently, it is recognized that EPR is a very heterogeneous phenomenon as it depends on the type of tumor and can vary significantly within the same tumor type (Lammers et al., 2012; Ojha et al., 2017). The degree of tumor vascularization and passive targeting of NNMs has been observed to be positively correlated (Theek et al., 2014). For example, Doxil® (pegylated liposomal doxorubicin) is the first FDA-approved NNM and has demonstrated superior efficacy in ovarian cancer and AIDS-related Kaposi's sarcoma compared to standard conventional therapies (Nichols and Bae, 2014). When doxorubicin is encapsulated within PEGylated liposomes, it delays and minimizes uptake and clearance by the reticuloendothelial system (RES), thereby prolonging circulation half-life. This allows the NNM to accumulate in the tumor tissue by exploiting the locally increased permeability of the tumor blood vessels, rather than in non-target healthy tissues which do not have such leaky vessels (Rahman et al., 2007). Furthermore, the use of pegylated liposomal doxorubicin avoids high plasma peak levels of free drug (Lyass et al., 2000) and significantly reduces the risk of cardiotoxicity by preventing doxorubicin release through the heart vasculature (Rahman et al., 2007).

### NNMs and active targeting

Active targeting, also termed ligand-targeting or receptor-mediated targeting, involves the use of ligands (e.g., antibodies, peptides or sugar moieties) which are physically or chemically conjugated onto the surface of NNMs to facilitate localization to and/or uptake by target cells (van der Meel et al., 2013; Danhier, 2016; Figure 1). Ligand-targeted NNMs have enormous potential for site-specific delivery of therapeutic compounds to designated cell types *in vivo*, which selectively express or over-express specific receptors (e.g., cellular receptors or cell adhesion molecules) at the site of disease (Willis and Forssen, 1998; Hua, 2013). For example, three sets of cellular targets are generally considered for active targeting in cancer—(i) targeting of cancer cells, which present overexpression of receptors for transferrin, folate, epidermal growth factor or glycoproteins; (ii) targeting of the tumor endothelium overexpressing vascular endothelial growth factors, integrins, vascular cell adhesion molecule-1 or matrix metalloproteinases; and (iii) targeting of stroma cells (e.g., macrophages, fibroblasts) that can acquire a tumor survival-promoting phenotype in response to cytokines in the tumor microenvironment (Coimbra et al., 2010; Danhier et al., 2010; Kuijpers et al., 2010; Danhier, 2016). There is still much debate about whether ligand-targeted NNMs are capable of significantly enhancing NNM accumulation at target sites over non-targeted NNMs (passive-targeting), with conflicting results reported in the literature (Ferrari, 2005; Puri et al., 2009; Riehemann et al., 2009; van der Meel et al., 2013). Enhanced therapeutic effects have been demonstrated with ligand-targeted NNMs, despite showing no differences in accumulation in target tissues compared to non-targeted NNMs. For example, similar high levels of tumor tissue accumulation were achieved with both non-targeted liposomes and liposomes conjugated with HER2 monoclonal antibody fragments (7–8% injected dose/g tumor tissue) in HER2-overexpressing breast cancer xenografts models (Kirpotin et al., 1997, 2006). However, significantly superior therapeutic results was demonstrated with the doxorubicin-loaded anti-HER2 immunoliposomes in comparison to all other control groups, including recombinant anti-HER2 Mab trastuzumab, non-targeted liposomal doxorubicin, and free doxorubicin (Park et al., 2002). Differences in pharmacodynamics of the targeted NNM formulation *in vivo* was suggested as the reason for the improved anti-tumor effect, by enhancing intracellular drug delivery to HER2-overexpressing cancer cells (Kirpotin et al., 2006).

### NNMs for triggered release

A third targeting strategy based on stimuli-responsive NNMs, referred to as triggered drug release, is currently receiving much attention from academia and industry. This class of NNMs is designed with the goal of enhancing drug release in tumors by means of endogenous or exogenous stimuli. Endogenous stimuli-responsive NNMs exploit factors associated with the local environment at the site of disease (Figure 1). For example, low pH, presence of redox gradients or certain enzymes in the tumor microenvironment. Exogenous-responsive NNMs respond to external stimuli to trigger drug release, such as temperature, light, magnetic field or ultrasound. Of these strategies, the use of an external hyperthermic trigger to release therapeutic compounds from NNMs (e.g., thermosensitive liposomal doxorubicin, ThermoDox®) appears to be the most promising to date (Needham et al., 2000). ThermoDox® was shown to be superior to its counterpart Doxil® in an *in vivo* model of non-resectable hepatocellular carcinoma (Torchilin, 2006; Sawant and Torchilin, 2012; Oude Blenke et al., 2013; Bertrand et al., 2014; Min et al., 2015; Jang et al., 2016; Shi et al., 2017). Thermosensitive liposomes are typically modified with temperature-sensitive lipids (e.g., distearoyl phosphocholine, DSPC) and/or polymers [e.g., poly(N-isopropylacrylamide)]. This composition allows the NNM to remain stable and retain their contents at physiologic temperatures, and undergo a phase change that makes them more permeable upon heating, thereby triggering the release of the cargo (Kono, 2001). The advantages of these NNMs can be further extended with the incorporation of imaging moieties (Figure 1) to enable monitoring of biodistribution, target accumulation and efficacy.

### NNMs approved and in clinical trials

A number of NNM products are on the market with more in clinical development. The majority of NNMs in clinical development incorporate already approved drugs and are based on a variety of drug delivery platforms, including polymeric micelles, liposomes, dendrimers, and inorganic nanoparticles (Torchilin, 2006; Wagner et al., 2006; Sercombe et al., 2015). Despite the arsenal of nanoparticulate targeted systems currently under preclinical development or in clinical trials, it is indisputable that liposomes are dominant on the NNM market (Table 1) and were the first FDA-approved NNM (Caster et al., 2017; Shi et al., 2017). In fact, liposomes have all the necessary features to allow formulation of highly toxic and/or poorly soluble drugs, such as paclitaxel and amphotericin B (Min et al., 2015; Caster et al., 2017). Soon after their discovery in 1965 (Sessa and Weissmann, 1968; Deamer, 2010), liposomes were proposed as drug delivery vehicles for both small molecules as well as macromolecular drugs (Gregoriadis and Ryman, 1971; Gregoriadis et al., 1971). Years of research led to the development of the first FDA-approved NNM (Doxil®/Caelyx®) as well as additional therapeutics (Allen and Cullis, 2013). Expectedly, many more NNMs are progressing to clinical investigation every year (Table 2), and again liposomal formulations represent the biggest share of the NNMs under clinical evaluation. The most frequently observed clinical benefit so far has been a reduction in toxicity with little evidence of improved efficacy. However, recently approved liposomal NNM, Vyxeos® (daunorubicin/cytarabine liposomal formulation), demonstrated improved survival and response rates, with tolerable toxicity in phase III clinical trials in older patients with therapy-related acute myeloid leukemia (t-AML) or AML with myelodysplasia-related changes (AML-MRC; Kim and Williams, 2018).

**Table 1:** NNM formulations currently approved for marketing.

**Type**	**Name**	**Drug**	**Indication**
Liposomal NNMs	Doxil/Caelyx	Doxorubicin	HIV-related Kaposi's Sarcoma, metastatic breast cancer, advanced ovarian cancer, multiple myeloma
	AmBisome	Amphotericin B	Fungal infections
	DaunoXome	Daunorubicin	HIV-related Kaposi's Sarcoma
	Myocet	Doxorubicin	Metastatic breast cancer
	Abelcet	Amphotericin B	Fungal infections
	Lipo-Dox	Doxorubicin	HIV-related Kaposi's Sarcoma, ovarian cancer, multiple myeloma
	Marqibo (Onco-TCS)	Vincristine	Adult AML
	Onivyde	Irinotecan	Pancreatic cancer
	Vyxeos (CPX-351)	Cytarabine and daunorubicin	AML
	Visudyne	Verteporfin	Wet AMD, myopia, ocular histoplasmosis
	DepoDur	Morphine	Postoperative analgesia
	DepoCyt	Cytarabine	Lymphomatous meningitis
Micellar NNMs	Genexol PM	Paclitaxel	Metastatic breast cancer, advanced lung cancer
	Nanoxel M	Paclitaxel	Advanced NSCLC, breast cancer, pancreatic cancer, ovarian cancer
Protein NNMs	Abraxane	Paclitaxel	Breast cancer, NSCLC, pancreatic cancer

*(Ref: ema.europa.eu; drugs.com; fda.gov)*.

**Table 2:** NNM formulations in clinical trials.

**Type**	**Name**	**Drug**	**Indication**	**Status**
Lipid NNMs	LiPlaCis	Cisplatin	Advanced or refractory solid tumors, metastatic breast cancer and skin cancer	Phase I/II
	ThermoDox	Doxorubicin	Hepatocellular carcinoma, breast cancer	Phase I/IIIII
	9NC-LP	9-Nitro-20 (S)-Camptothecin	Ewing's sarcoma and other solid tumors with lung involvement, endometrial cancer	Phase I/II completed
	SPI-077	Cisplatin	Ovarian cancer, relapsed/progressive osteosarcoma metastatic to the lung	Phase I/ II/ III
	Lipoxal	Oxaliplatin	Colorectal cancer, glioma	Phase II
	EndoTAG-1	Paclitaxel	Pancreatic cancer, liver metastases, HER2 and triple negative breast cancer	Phase II completed
	OSI-211	Lurtotecan	SCLC	Phase I/II completed
	LE-DT	Docetaxel	Solid tumors, pancreatic cancer	Phase I/II completed
	LEP-ETU	Paclitaxel	Breast cancer, neoplasm, gastric carcinoma	Phase I/II/IV
	TKM-080301	siRNA against PLK1	Advanced hepatocellular carcinoma, solid tumors or lymphomas that are refractory to conventional therapies; colorectal, gastric, breast and ovarian cancers with hepatic metastases	Phase I/II completed
	Atu027	siRNA against PKN3	Advanced solid tumors, pancreatic cancer	Phase I/II completed
	2B3-101	Doxorubicin	Advanced solid tumors, brain metastases, lung and breast cancers, melanoma, malignant glioma	Phase I/II completed
	MTL-CEBPA	saRNA	Liver cancer	Phase I
	TLI	Topotecan	SCLC, ovarian cancer, solid tumors	Phase I
	MM-398 Onivyde	Irinotecan	Solid tumors, ER/PR positive and triple negative breast cancer, metastatic breast cancer with active brain metastasis, SCLC, metastatic pancreatic cancer	Phase I/II/III
	MM-302	Doxorubicin	Breast cancer	Phase I
	ATI-1123	Docetaxel	Advanced solid tumors	Phase I completed
	SGT-53	p53 pDNA	Solid tumors, recurrent glioblastoma	Phase I/II
	SGT-94	RB94 pDNA	Solid tumors, recurrent glioblastoma	Phase I, Phase II
	Anti-EGFR-IL-DOX	Doxorubicin	Solid tumors	Phase II
	RNL	Rhenium-186	Glioblastoma and astrocytoma (treatment and imaging)	Phase I/II
	Patisiran	siRNA	TTR-mediated amyloidosis	Phase I/II/III
Polymeric NNMs	Paclical	Paclitaxel	Ovarian cancer	Phase III completed
	NK105	Paclitaxel	Gastric cancer	Phase III completed
	BIND-014	Docetaxel	NSCLC, solid tumors	Phase II completed
	CALAA-01	RRM2 siRNA	Solid tumors	Phase II terminated
	CRLX101	Camptothecin	NSCLC	Phase II completed

*(Ref: clinicaltrials.gov)*.

## Challenges in the clinical translation of nanomedicines

The clinical translation of NNMs is an expensive and time-consuming process. NNM technology is usually far more complex in comparison to conventional formulation technology containing free drug dispersed in a base (e.g., tablets, capsules and injections; Teli et al., 2010; Tinkle et al., 2014; Sainz et al., 2015). Key issues related to the clinical development of NNMs are listed in Table 3, and include biological challenges, large-scale manufacturing, biocompatibility and safety, intellectual property (IP), government regulations, and overall cost-effectiveness in comparison to current therapies (Allen and Cullis, 2004, 2013; Zhang et al., 2008; Sawant and Torchilin, 2012; Narang et al., 2013). These factors can impose significant hurdles limiting the appearance of NNMs on the market, irrelevant of whether they are therapeutically efficacious or not.

**Table 3:** Considerations for the translational development of nanomedicines.

**NANOPHARMACEUTICAL DESIGN**
***Key Considerations*** ■ Route of administration ■ Reduce complexity in formulation design ■ Final dosage form for human use ■ Biocompatibility and biodegradability ■ Pharmaceutical stability (physical and chemical)
***Current Obstacles***
■ Large-scale production according to GMP standards ◦ *E.g., Reproducibility, infrastructure, techniques, expertise and cost* ■ Quality control assays for characterization ◦ *E.g., Size and polydispersity, morphology, charge, encapsulation, surface modifications, purity and stability*
**PRECLINICAL EVALUATION**
***Key Considerations*** ■ Need for validated and standardized assays for early detection of toxicity ■ Evaluation in appropriate animal models of disease ■ Adequate understanding of *in vivo* behavior, incl. cellular and molecular interactions ◦ *Pharmacokinetics (absorption, distribution, metabolism and excretion)* ◦ *Pharmacodynamics (intracellular trafficking, functionality, toxicity and degradation)*
***Current Obstacles***
■ Development of more specialized toxicology studies for nanomedicines ■ Adequate understanding of the interaction of NNM with tissues and cells ■ Adequate structural stability of NNM following *in vivo* administration ■ Limited degree of accumulation of nanomedicines in target organs/tissues/cells
**CLINICAL EVALUATION FOR COMMERCIALIZATION**
***Key Considerations*** ■ Simplification of development pathways from invention to commercialization to minimize time and expense ■ Evaluation of safety/toxicity in humans (acute and chronic) ■ Evaluation of therapeutic efficacy in patients ■ Optimal clinical trial design
***Current Obstacles***
■ Lack of clear regulatory guidelines specific for NNMs ■ Complexity of NNM patents and IP ■ Limited understanding of the biological interaction of NNM with the biological environment (incl. target site) in the body of patients

### Biological challenges

Traditionally, NNM development has been based on a formulation-driven approach, whereby novel delivery systems are firstly engineered and characterized from a physicochemical perspective. It is only when attempting to align the NNM with a pathological application that limitations in the clinical translation of the system have been identified. Understanding the relationship between biology and technology, including understanding the influence of disease pathophysiology on nanomedicine accumulation, distribution, retention and efficacy, as well as the biopharmaceutical correlation between delivery system properties and *in vivo* behavior in animals versus humans are important determinants for the successful translation of NNMs. Therefore, applying a disease-driven approach by designing and developing NNMs that are able to exploit pathophysiological changes in disease biology has been suggested to improve clinical translation (Hare et al., 2017).

From the outset in NNM development, it is essential to consider the relationship between disease pathophysiology and the heterogeneity of the disease in humans, and the importance of physicochemical characteristics of different NNMs to overcoming biological barriers to enable improved targeting to diseased tissue and/or reduced accumulation in non-target organs. Considerably less research effort has been dedicated to comprehensively understanding the correlations between NNM behavior and patient biology in specific clinical applications as well as disease heterogeneity in patients—which are likely the major reasons for the failure seen in the translation of promising NNMs in clinical trials (Hare et al., 2017). These biological challenges can be a significant deterrent for pharmaceutical industry investment into nanomedicines. In order to reduce investment risk for NNMs, the preclinical data sets need to comprehensively evaluate therapeutic efficacy, safety, biodistribution, and pharmacokinetics in appropriate animal models of the disease that are relevant to human disease. Evaluation of NNMs in multiple preclinical animal models that represent aspects of the clinical disease is preferred to achieve reproducibility of results for the specific disease and not for a specific animal model. In addition, animal models that reflect only a narrow spectrum of the clinical disease may provide useful data that can predict their suitability for treating a specific patient sub-group (Hare et al., 2017). Differences in the anatomy and/or physiology of the animal species compared to humans should be taken into account based on different routes of administration. Preclinical studies of NNMs should also be conducted under appropriate randomization and blinding to reduce bias, as well be evaluated against proper controls, including the gold standard treatment and not just free drug solution. These factors are currently lacking in many published studies, which makes it difficult to assess clinical applicability and translatability. Other considerations include designing preclinical studies to optimize NNM performance *in vivo*, dosing schedules, and treatment combinations based on the specific clinical disease, as well as understanding the influence of disease progression and severity on nanomedicine performance. This will determine whether specific patient sub-groups may respond more favorably to NNM-based treatment.

Interestingly, the majority of the NNM formulations in development and clinical trials are focused on cancer targeting, including more than 80% of the publications on nanomedicine in the last two decades alone (Park, 2017). Despite the large number of publications, the translation of the published studies to clinical applications has been disappointing. Cancer targeting of NNMs has generally been universally based on the EPR effect, despite the fact that EPR-mediated accumulation has only been reported for some tumor types (Maeda, 2015). Tumors, like other clinical diseases, can be highly heterogeneous and can show inter-patient and intra-patient variability as the disease progresses. Hence a one-size-fits-all approach when designing NNM-based treatment is unlikely to translate to clinically beneficial outcomes. The EPR effect has increasingly been exploited for NNM targeting in other non-cancer conditions, especially those involving an inflammatory component that causes leakiness of inflamed blood vessels (e.g., rheumatoid arthritis, atherosclerosis, and inflammatory bowel disease; Metselaar et al., 2003, 2004; Maiseyeu et al., 2009; Crielaard et al., 2012; Hua and Cabot, 2013; Hua et al., 2015; Milane and Amiji, 2017). It should be appreciated that not all diseases can be accessed with NNMs due to biological barriers and that the EPR effect is unlikely to be present in all clinical diseases. EPR is also not the only determinant of NNM efficacy. NNM activity is also influenced by the extent of cellular uptake and kinetics of drug release within target tissues (Hare et al., 2017).

Furthermore, the advantages of ligand-targeted NNMs in the clinical research phase have so far been negligible, despite the enhanced accumulation in target sites and therapeutic outcomes in a number of preclinical studies (Sercombe et al., 2015). Potential reasons for this discrepancy have previously been reviewed (Sawant and Torchilin, 2012; Allen and Cullis, 2013), and include factors such as target accessibility and expression, disease-dependent anatomical and physiological barriers, and formulation stability. In addition, the optimal targeting ligand density on the surface of each NNM has yet to be determined and will likely depend on characteristics of the molecular target (e.g., expression, location, internalization rate and immunogenicity; Puri et al., 2009; Hua and Wu, 2013; Kraft et al., 2014). Detailed analysis of the degree of NNM accumulation, cellular internalization, intracellular functionality and intracellular degradation will also be important considerations for clinical validation and translation (Puri et al., 2009). Through extensive experimentation, we are gaining a better understanding of the more appropriate clinical applications for ligand-targeted NNMs. Therefore, by taking a disease-driven approach to NNM development, it will be possible to build comprehensive preclinical data sets that best predict efficacy for patient sub-groups and support translatable clinical development.

### Large-scale manufacturing

One of the important factors contributing to the slow pace in the clinical translation of NNMs is the structural and physicochemical complexity of the formulation itself. Platforms that require complex and/or laborious synthesis procedures generally have limited clinical translation potential, as they can be quite problematic to pharmaceutically manufacture on a large-scale (Teli et al., 2010; Tinkle et al., 2014; Barz et al., 2015; Sainz et al., 2015). Pharmaceutical manufacturing development is centered on quality and cost. Quality includes the manufacturing process and stability of the formulation, with NNM manufacturing being challenged by potential issues related to: (i) poor quality control; (ii) scalability complexities; (iii) incomplete purification from contaminants (e.g., by-products and starting materials); (iv) high material and/or manufacturing costs; (v) low production yield; (vi) insufficient batch-to-batch reproducibility, consistency and storage stability of the final product (e.g., regarding size distribution, porosity, charge and mass); (vii) lack of infrastructure and/or in-house expertise; (viii) chemical instability or denaturation of the encapsulated compound during the manufacturing process; and (ix) scarcity of venture funds and pharmaceutical industry investment (Teli et al., 2010; Narang et al., 2013; Hafner et al., 2014; Tinkle et al., 2014).

An essential requirement for clinical translation is to have access to a preparation method that allows the production of large scalable quantities of NNMs, which is also consistently manufactured at the same high level of quality and batch-to-batch reproducibility to set specifications (Grainger, 2013; Lammers, 2013; Barz et al., 2015). Suitable methods for the industrial scale production of several basic nanomedicine platforms, such as liposomes, have been successfully developed without the need for numerous manufacturing steps or the use of organic solvents (Jaafar-Maalej et al., 2012; Kraft et al., 2014). The challenges arise when the NNM system becomes more complex. For example, with the addition of surface modification with coatings and/or ligands, inclusion of multiple targeting components, or by encapsulating more than one therapeutic agent. Integration of multiple components into a single nanosized carrier requires multiple steps in the production process, which inevitably poses problems for large-scale good manufacturing (cGMP) production, increases the cost of production, and makes the quality assurance and quality control (QA and QC) evaluation of such products more difficult (Teli et al., 2010; Svenson, 2012; Tinkle et al., 2014).

Characteristics of the manufactured NNM need to be well-defined and reproducibly generated to allow initiation of clinical translation. Chemistry, Manufacturing, and Controls (CMC) information is required for investigational new drugs (IND) at each phase of investigation to ensure proper identity, strength or potency, quality, and purity of the drug substance and drug product (FDA, 2003). The type of information submitted will depend on the phase of the investigation, the extent of the human study, the duration of the investigation, the nature and source of the drug substance, and the drug product dosage form (FDA, 2003). The characterization and validation of more complex NNMs can be particularly challenging due to the sheer number of parameters to address (e.g., size distribution, morphology, charge, purity, drug encapsulation efficiency, coating efficiency, and density of conjugated ligand/s; Teli et al., 2010). Batch-to-batch variation of NNMs can potentially lead to significant changes to their physicochemical properties (e.g., polarity and size), pharmacokinetic parameters (i.e., absorption, distribution, metabolism and excretion), and/or pharmacodynamic interactions (e.g., cellular interaction and activity; Teli et al., 2010; Tinkle et al., 2014; Barz et al., 2015). In addition, NNMs need to be stable after the manufacturing process, during long-term storage, and upon clinical administration (i.e., to avoid massive drug release or aggregation in the bloodstream en route to the site of action).

### Biocompatibility and safety

Detailed toxicology is essential for the clinical translation of NNMs to determine the overall safety for human use (Nystrom and Fadeel, 2012). Pharmaceutical regulatory authorities generally recommend that the sponsor carefully assess for any changes in the drug substance and drug product manufacturing process or drug product formulation at any phase of clinical development, in order to determine if the changes can directly or indirectly affect the safety of the product. CMC modifications throughout the IND process that can affect safety include: (i) changes in the synthetic pathway or reagents used to manufacture the drug substance, product or formulation; (ii) changes resulting in a different impurity profile; (iii) changes in the actual manufacturing method (e.g., chemical synthesis, fermentation, or derivation from a natural source); (iv) changes in the source material; (v) changes in the method of sterilization of the drug substance or drug product; (vi) changes in the route of administration; (vii) changes in the composition and/or dosage form of the drug product; (viii) changes in the drug product manufacturing process that can affect product quality; and (ix) changes in the drug product container closure system that can affect product quality (e.g., dose delivery; FDA, 2003). If any changes are identified, stringent procedures are in place to ensure appropriate comparison testing of the drug substance and/or drug product produced from the previous manufacturing process with the changed manufacturing process to evaluate product equivalency, quality, and safety (FDA, 2003). When analytical data demonstrate that the materials manufactured before and after are not comparable, sponsors should perform additional qualification and/or bridging studies to support the safety and bioavailability of the material to be used in the proposed trials (FDA, 2003).

Knowledge of the activity and toxicities of the free drug, the behavior of different NNM delivery systems and their interaction with biological components, and the influence of drug release rate on target and off-target concentrations of bioavailable drug allow the ability to predict potential side effects or toxicities *in vivo* (Hare et al., 2017). In particular, the rational design of NNMs from the early phase of material selection, production method optimization, and product purification is of fundamental importance to increase their clinical translation potential (Accomasso et al., 2018). Although the safety of some common materials such as phospholipids and biodegradable polymers have been studied previously (Storm et al., 1993), increasing the complexity of NNMs, such as the use of different synthetic compositions, coatings and ligands, can have a significant effect on the biocompatibility, biodistribution and toxicology profile of nanomedicines following *in vivo* administration (Allen and Cullis, 2004, 2013; Zhang et al., 2008; Sawant and Torchilin, 2012; Narang et al., 2013; Tinkle et al., 2014). For example, complement activation-related pseudoallergy (CARPA) is an acute adverse immune reaction caused by many NNMs (Szebeni, 2005; Sercombe et al., 2015; Szebeni and Storm, 2015; Jackman et al., 2016). The complement system is part of the innate immune response and is involved in a range of inflammatory and immunological processes (Moghimi and Hunter, 2001). CARPA is an immediate, non-IgE-mediated hypersensitivity reaction that can cause symptoms, including anaphylaxis, facial swelling, facial flushing, chills, headache, and cardiopulmonary distress (Szebeni, 2005). This adverse reaction is generally managed by slowing the infusion rate or ceasing therapy, as well as the use of standard allergy medications (e.g., antihistamines, corticosteroids and epinephrine; Sercombe et al., 2015; Szebeni and Storm, 2015). The development of immunogenic reactions to NNM-based therapies may lead to altered pharmacokinetics, loss of efficacy, and the rise of potentially serious toxicities (e.g., anaphylaxis; Szebeni and Moghimi, 2009; Szebeni and Storm, 2015).

There is a regulatory need for validated, sensitive and standardizable assays incorporating *in vitro, ex vivo* and *in vivo* protocols to appropriately assess the nanotoxicology of NNMs during the early stages of clinical development (Dobrovolskaia and McNeil, 2013; Jackman et al., 2016; Accomasso et al., 2018). Comprehensive *in vitro* or *ex vivo* assays for nanosafety testing are essential to screen for potential hazards prior to preclinical evaluation in animal models (Gaspar, 2007). For example, standardized *in vitro* protocols using different cell culture models (i.e., blood, liver, lung, brain, placenta, gastrointestinal system) to assess potential risk of cytotoxicity, immunotoxicity, and genotoxicity of NNMs (Accomasso et al., 2018). This is particularly important with the development of NNMs incorporating many new materials with the goal for use in the clinical setting. In order to do this effectively across the board, standardized reference materials would need to be established and the testing would also need to be relevant for the intended route of administration (Tinkle et al., 2014). Although current testing approaches are limited and insufficient for nanotoxicology evaluations for clinical translation, a number of techniques that are more specific for nanomedicines are under development. This includes alternative test strategies, high-throughput screening techniques, high-content screening, and computational modeling (Nel et al., 2013; Oomen et al., 2014; Dusinska et al., 2015; Accomasso et al., 2018). These techniques have the potential to analyze in a comparative way many NNMs simultaneously.

There is also a need to perform specialized toxicology studies in animal models to assess both short-term and long-term toxicity, as circulation half-lives and drug retention times are generally significantly increased with nanoencapsulation. A thorough understanding of the absorption, distribution, metabolism, and excretion of emerging nanomaterials *in vivo* is important to predict the toxicological responses to NNMs (Dobrovolskaia and McNeil, 2013; Tinkle et al., 2014). Adequate assessment protocols are needed to monitor various aspects of the NNM drug delivery process, including pharmacokinetics, biodistribution, target site accumulation, local distribution at the target site, localization in healthy tissues, kinetics of drug release, and therapeutic efficacy (Kunjachan et al., 2015). Incorporation of real-time imaging techniques have enabled better understanding of the interaction of NNMs with biological organs and tissues following *in vivo* administration (Gaspar, 2007; Nystrom and Fadeel, 2012; Dobrovolskaia and McNeil, 2013; Kunjachan et al., 2015).

In addition, biocompatibility, immunotoxicological, and inflammatory potential should be assessed, with functional outcomes correlated with mechanisms of tissue uptake and clearance (Gaspar, 2007). These parameters need to be well-investigated based on dose, dosage form and route of administration to establish safe limits prior to clinical trials (Gaspar, 2007; Nystrom and Fadeel, 2012). This is of particular importance for NNMs composed of materials that have never been used before in clinical applications. Even in the clinical trial phase, regulatory protocols should be in place to detect any toxicity caused not only by the encapsulated therapeutic compounds, but also novel mechanisms unique to nanotechnology (Gaspar, 2007; Nystrom and Fadeel, 2012). For example, short- and long-term effects of NNM accumulation in RES organs (esp. liver, kidneys, spleen, lungs, lymph nodes, and bone marrow; Senior, 1987; Szebeni and Barenholz, 2009; Szebeni and Moghimi, 2009), which are the main sites for NNM accumulation following systemic administration (Poste et al., 1976; Senior, 1987). The cells of the RES are also part of the innate immune system, which has raised concerns regarding whether macrophage saturation by NNMs can cause immunosuppression and increase the risk of infections (Sercombe et al., 2015; Liu et al., 2017). There have been no reports of clinically significant immunosuppression at therapeutic doses of non-cytotoxic NNMs, despite suggestions that excessive NNM deposition in macrophages may impair their phagocytic capacity or modulate other cellular functions (Szebeni and Barenholz, 2009; Szebeni and Moghimi, 2009). However, NNMs that contain cytotoxic compounds are capable of inducing macrophage destruction following uptake (Szebeni and Barenholz, 2009; Szebeni and Moghimi, 2009), with indirect signs that suggest the possibility of some immune suppression (Storm et al., 1998; Szebeni and Barenholz, 2009; Szebeni and Moghimi, 2009). For example, administration of Doxil® in mice was reported to interfere with the clearance of bacteria from the blood due to macrophage suppression (Storm et al., 1998; Szebeni and Barenholz, 2009). Addressing these issues are necessary to safeguard the application of emerging NNMs in the clinical setting.

### Intellectual property (IP)

Given the complexities of incorporating nanotechnology into biomedical and clinical applications, there needs to be more precise definitions on what constitutes novel IP of a nanomedicine (Satalkar et al., 2015). Nanomedicines are complex as they have a number of variable components, and bridge between the field of medicine and medical device (Paradise et al., 2009). Generally, the control of a NNM product requires an IP position on: (i) the encapsulated cargo; (ii) the carrier technology; and (iii) the characteristics of the drug and carrier together. Although this definition is straightforward, it does open up a number of problems with the issuing of patents to date (Bawa, 2007; Bawa et al., 2008). For example, NNMs that incorporate existing drugs with novel carrier technology, or those that incorporate existing drugs with existing carrier technology for a new biomedical or disease application. The IP situation becomes even more confusing with more complex drug delivery systems, such as those which incorporate commercially available targeting ligands (e.g., antibodies) or coatings (e.g., Eudragit®) that are owned by other companies. IP strategies may likely involve multiple patents associated with any given technology and the need for cross-licensing arrangements (Murday et al., 2009). Therefore, new IP practices and protocols are required to simplify the pathway from invention to commercialization to reduce the time and expense required for negotiating collaboration and licensing agreements (Murday et al., 2009).

With the significant increase in the number of nanotechnology patent applications over the last few decades, other key issues that need to be addressed include patent review delays, patent thickets, and issuance of invalid patents (Bawa, 2005, 2007; Bawa et al., 2005). There needs to be a universal nano-nomenclature on identical or similar nanostructures or nanomaterials, and more refined search tools and commercial databases to avoid the issuing of multiple nanopatents on the same invention (Bawa et al., 2005; Bawa, 2007). Databases used by the Patent and Trademark Office (PTO) need to be able to search through nanotech-related prior art that resided in scientific publications world-wide, including earlier publications that preceded the emergence of online publication databases (Tinkle et al., 2014). Patent examiners also require expertise and training with respect to the emerging fields of nanotechnology and nanomedicine. The complexities with nanotechnology have led to the so called “patent thickets”, which can lead to costly litigation and halt commercialization efforts (Tinkle et al., 2014). Therefore, improved clarity on IP and patenting surrounding nanotechnology in health and medicine is required, and will need to involve implementation of universal regulations and policies that are tailored toward this niche commercialization field.

### Government regulations

Nanomedicines have significant potential to increase the growth of the pharmaceutical market and improve health benefits, however the current scientific and regulatory gap for nanomedicines is large and challenging. Commercialization of nanomedicines is highly dependent on a number of regulatory factors based on government policies in the area of manufacturing practice, quality control, safety, and patent protection (Gaspar, 2007; Tinkle et al., 2014; Sainz et al., 2015). The lack of clear regulatory and safety guidelines has affected the development of NNM products toward timely and effective clinical translation (Gaspar, 2007; Tinkle et al., 2014; Sainz et al., 2015). For example, polymers have been widely investigated as an effective platform for NNM strategies; however, their safety and efficacy is highly dependent on the polymer molecular weight, polydispersity, molecular structure, and conjugation chemistry (Gaspar and Duncan, 2009; Diab et al., 2012). Due to the increased number of novel polymeric materials and complex polymeric-based NNM formulations, there is an urgent need for an appropriate regulatory framework to assist in evaluation (Gaspar and Duncan, 2009). As each polymer-based NNM is different, it is important to consider each individually based on doses, administration routes, dosing frequency, and proposed clinical use. This would be the same for most other NNM platforms.

NNMs are currently regulated within the conventional framework governed by the key regulatory authority of each country (e.g., FDA, TGA, and EMA). Although NNMs have been on the market for nearly two decades, the first generation of NNM products passed regulatory approval by only having to meet general standards, applicable to medicinal compounds. These regulations are no longer appropriate to confirm the quality, safety, and efficacy of NNMs for clinical use (Gaspar, 2007; Tinkle et al., 2014; Sainz et al., 2015). Reasons for this are based on the complex structure of NNMs, their unclear interaction with cells and tissues within the human body, increased complexity of clinical use, and the multifunctional nature of some formulations (e.g., integration of therapeutics with imaging diagnostics; Gaspar, 2007; Tinkle et al., 2014; Sainz et al., 2015). Regulatory standards and protocols validated specifically for nanoparticles are needed that bridge both medicine and medical devices regulations. This should take into account a NNM's complexity, route of administration, pharmacokinetics, pharmacodynamics and safety profile, as well as provide information on the most appropriate clinical trial design and patient selection (Tinkle et al., 2014). There needs to be a fine balance to ensure the safety and quality of NNMs without over-regulation, which can negatively affect the progress of innovative products to the market, by escalating costs for achieving regulatory approval and/or consuming a significant portion of the life of a patent.

Development of global regulatory standards for NNMs should be established alongside key countries with invested interest. Although major steps have been taken in the last 5 years, a closer collaboration between regulatory agencies, academia, research and industry is needed (Gaspar, 2007; Murday et al., 2009; Hafner et al., 2014). This is of particular importance due to the limited availability of contract manufacturing organizations world-wide that specialize in producing NNM products in accordance with the requirements for good manufacturing practice (GMP; Hafner et al., 2014). It should be noted that this limited number of manufacturing organizations may be further divided based on their infrastructure capabilities of producing specific NNM platforms (e.g., liposomes, polymeric nanoparticles, dendrimers and drug-polymer conjugates). Therefore, NNMs produced in these manufacturing organizations will likely be marketed in multiple countries and thus should be governed under the same regulatory standards (Hafner et al., 2014). There will need to be complete evaluation and documentation of production processes for NNMs, incorporating appropriate industrial standards for both quality control and prevention of environmental issues (Gaspar, 2007). Manufactured NNMs will still need to meet general pharmaceutical standards such as purity, sterility, stability, manufacturing operations, and related industrial control standards (Gaspar, 2007). In addition, new analytical tools and standardized methods will need to be implemented to evaluate key physical characteristics of NNMs that can affect *in vivo* performance such as particle size and size distribution, surface chemistry, morphology, surface area, surface coating, hydrophilicity, porosity, and surface charge density (Gaspar, 2007; Tinkle et al., 2014; Sainz et al., 2015). These methods will vary for different nanomaterials and nanostructures. Thus, regulatory authorities should work together to develop the testing methods and appropriate standardized protocols for toxicity studies and regulatory requirements, which will be needed to ensure the efficacy and safety of current and emerging NNMs.

## Perspectives on the translational development of nanomedicines

From a therapeutic perspective, increasing drug accumulation at target tissues and minimizing systemic adverse effects are still the biggest design challenges to meet when developing new drug delivery systems. Even though promising NNMs may demonstrate significant efficacy in *in vitro* or *ex vivo* studies, it is important to evaluate the platforms *in vivo* using appropriate animal models of the disease. It is here where many of the current NNM platforms have shown limited specificity, accumulation and/or stability, therefore providing unsatisfactory results to warrant progression in the R&D process (Hua et al., 2015). Efficacy in an animal model also does not necessarily equate to efficacy in humans, as drug delivery within the human body is complex and can be highly variable, especially when associated with disease (Hare et al., 2017). Therefore, this concept of designing nanomedicines that act like a “magic bullet,” which refers to the exclusive delivery of active compounds to specific organs, tissues or cells, is just not realistic when taking into account the pharmacokinetic and pharmacodynamic processes that occur following administration into the body (Barz et al., 2015). This term should refer to the development of realistic therapeutic platforms, in which therapeutic effects are maximized, doses are minimized, and complexity in dosage form design is reduced (Barz et al., 2015).

Complexity in dosage form design is a key factor in the ability for a NNM formulation to be translated to the clinic, irrelevant of its therapeutic efficacy. Simplification in formulation design is required to allow efficient and reproducible large-scale manufacturing (Grainger, 2013; Lammers, 2013; Barz et al., 2015). Any added complexities to the basic NNM platform would need to show significantly improved benefits that is reliable and reproducible in animal models and patients, due to the added costs and complexity in the manufacturing process. For example, further studies are required to examine the benefits of ligand-targeted delivery systems over basic NNM platforms, in particular the reliability and consistency of the expression of the target across disease severity and in different patients (Hua et al., 2015; Sercombe et al., 2015; Hare et al., 2017). In addition, when translating findings from animal models to humans, we need to determine how to modify these formulations so that they are appropriate for human administration (Hua et al., 2015). *In vivo* studies are typically conducted in animal models of experimental diseases, especially in mice and rats, which can place limitations on the size and consistency of the dosage form that can be administered—for example, via oral, topical or intraperitoneal delivery (Hua et al., 2015; Sercombe et al., 2015). The practicability of designing dosage forms that are both acceptable to humans and efficacious should be further explored for clinical studies. Thus, there needs to be a balance between complexity, therapeutic efficacy, and clinical translation.

To transition NNMs to the clinic, attention should be given to nanosized carriers that are stable following *in vivo* administration, easily able to be up-scaled for manufacturing with high control over their physicochemical properties (e.g., size and polydispersity, morphology, drug encapsulation efficiency, and charge), as well as being composed of materials that are biocompatible, biodegradable, and non-toxic. As nanoparticles are able to enter cells and interfere with molecular pathways, synthetic polymers and lipids should be carefully evaluated for potential short-term and long-term toxicity for clinical application (Gaspar and Duncan, 2009). For example, potentially toxic *in vitro* and *in vivo* effects have been identified with the use of cationic polymers and lipids, including reduced number of mitoses, cell shrinking, detrimental effects on key cellular proteins (e.g., protein kinase C), and vacuolization of the cytoplasm (Lv et al., 2006).

## Pathway to translation and commercialization

The experimental development of NNMs is progressing at a fast pace, however significant challenges still exist in promoting these platforms into clinically feasible therapies (Table 3). The majority of NNMs in the clinic are for the treatment of cancer, predominantly by the parenteral route of administration. They are structurally based on simple nanomedicine platforms, in particular basic nanoparticles, surface charge-modified nanoparticles, and PEGylated nanoparticles (Hafner et al., 2014; Sainz et al., 2015). Although clinical applications of nanotechnology for non-cancer diseases are increasing based on promising experimental results, there are several barriers that have slowed progress in the preclinical and, especially, clinical stages of development. This includes issues surrounding complexity in manufacturing and characterization, lack of understanding of *in vivo* pharmacokinetics and pharmacodynamics, acute and chronic toxicity, and cost-effectiveness (Gaspar, 2007; Teli et al., 2010; Hafner et al., 2014; Tinkle et al., 2014; Sainz et al., 2015). These challenges are even greater with increasing complexity of the NNM design.

The pace for the clinical translation of NNMs has been relatively slow as the development trajectory is very costly, complex and time-consuming, which has affected the attitudes of the pharmaceutical industry and capital investors. There has to be a clear positive benefit-to-risk ratio that will accompany the clinical implementation of products and procedures based on nanotechnology. In particular, the cost-benefit analysis may be a limitation to the clinical translation of some NNMs when compared to an approved counterpart or existing therapies. This analysis has to be clear before starting the development process. Emerging NNM products, which are more complex in structure and more expensive than conventional therapies, need to provide an overall reduction in health care costs and provide a worthwhile opportunity for the pharmaceutical industry to invest its R&D budgets (Hafner et al., 2014). This reduction in health care costs is likely to be obtained by increasing therapeutic efficacy, improving quality of life, reducing adverse effects or toxicities in non-target organs, and/or reducing the need for surgical or other high-risk interventions (Gandjour and Chernyak, 2011). Nanopharmaceuticals can offer the ability to extend the economic life of proprietary drugs and create additional revenue streams (Tinkle et al., 2014). In addition, market analysis, investment risk, potential profit margins, and value proposition of novel NNMs are important factors for the pharmaceutical industry and investors. Typically, pharmaceutical products that are developed to address larger disease populations with treatment expected in a primary or secondary care setting are preferred by the pharmaceutical industry. From a business perspective, the necessary infrastructure, understanding of NNMs, and skill set required for the commercial development of NNMs are not currently well represented at most pharmaceutical companies. These factors should be taken into account when assessing the overall cost-effectiveness of NNMs in comparison to existing therapies.

Nanomedicines generally face a number of regulatory approval hurdles. The control of materials in the nanosize range often presents greater scientific and technical challenges compared to conventional formulations (Gaspar, 2007; Teli et al., 2010; Hafner et al., 2014; Tinkle et al., 2014; Sainz et al., 2015). NNMs encompass a number of different types of nanomaterials and nanostructures, which make it even more challenging to establish appropriate regulatory protocols and tools to ensure standardized GMP manufacturing and characterization, safety and toxicology evaluation, and clinical trial design. These procedures are paramount to confirming therapeutic efficacy and safety prior to marketing approval for use in patients on a larger scale. Effective clinical translation will require an interdisciplinary approach to develop novel protocols, assays and infrastructure for the manufacturing and characterization of NNMs (Gaspar, 2007; Teli et al., 2010; Hafner et al., 2014; Tinkle et al., 2014; Sainz et al., 2015). This will need to involve experts from academia and industry with specialty in pharmaceutics, engineering, biology, medicine, and toxicology. Potential approaches to fast-track promising novel NNMs to clinical trials include the establishment or coordination of laboratories and centers that have expertise in (i) characterizing NNM platforms, (ii) conducting preclinical studies on NNMs for submission to regulatory agencies, (iii) scale up laboratory preparation of nanomaterials according to regulatory and industry standards for early clinical trials, and (iv) designing and conducting clinical trials of NNM platforms (Hafner et al., 2014).

## Conclusion

Overall, the use of nanotechnology in medicine has the potential to have a major impact on human health. It has been suggested to facilitate the development of personalized medicine for specific patient sub-groups, in which therapy is tailored by the patient's individual genetic and disease profile (Teli et al., 2010; Mura and Couvreur, 2012; Laroui et al., 2013). For example, disease-specific characteristics such as capillary permeability (Calcagno et al., 2015), cellular receptor expression and molecular pathway activation could be analyzed and used to design personalized nanomedicines (Teli et al., 2010; Mura and Couvreur, 2012; Laroui et al., 2013). The physicochemical properties (e.g., size and structure) of the delivery system can also be modified according to the severity of the disease for optimal therapeutic benefits (Hua et al., 2015). This concept would significantly advance the way in which we treat patients. However, for this to occur, there are still a number of issues that need to be addressed as detailed in this review—from our basic understanding of the biology of specific diseases and the biological interaction of NNMs in patients, to commercialization hurdles related to manufacturing, costs, and regulatory standards. Finally, researchers need to consider minimizing the complexity of NNMs and take into account the final dosage form for human use, in order for a formulation to have the potential to be translated into a clinically applicable therapeutic. Reducing complexity to the minimum required for pathophysiological or medical need is paramount in nanoparticle design and synthesis to generate clinically translatable nanosized therapeutics.

## Author contributions

All authors listed have made a substantial, direct and intellectual contribution to the work, and approved it for publication.

### Conflict of interest statement

The authors declare that the research was conducted in the absence of any commercial or financial relationships that could be construed as a potential conflict of interest.
